# Analyzing Self-Efficacy and Summary Feedback in Automated Social Skills Training

**DOI:** 10.1109/OJEMB.2021.3075567

**Published:** 2021-04-27

**Authors:** Hiroki Tanaka, Hidemi Iwasaka, Yasuhiro Matsuda, Kosuke Okazaki, Satoshi Nakamura

**Affiliations:** Nara Institute of Science and Technology 12708 Ikoma-shi Nara 630-0192 Japan; Nara Medical University 12967 Kashihara-shi Nara 634-8521 Japan

**Keywords:** Embodied conversational agents, self-efficacy, social skills training, summary feedback

## Abstract

*Goal:* Although automated social skills training has been proposed to enhance human social skills, the following two aspects have not been adequately explored: what types of feedback are effective from virtual agents and the extent to which such systems enhance users' social self-efficacy. *Methods:* We developed an automated social skills trainer+ that follows human-based social skills training processes and implemented two types of feedback: 1) a summary of the displayed feedback and 2) feedback based on the results of their previous training. Using our developed system, we measured social self-efficacy, feedback evaluations, and the third-party ratings of participants between pre- and post-training as well as their social responsiveness scales. *Results:* Self-efficacy is significantly correlated to the social responsiveness scale (r = −0.72) and can be improved with our system (mean improvement of 0.68, p < 0.05). The participants highly rated the feedback that was compared to their past training (14 out of 16, p < 0.05) more than the cases without it and the displayed summary feedback (11 out of 16, p = 0.21) more than the verbal comments. *Conclusions:* Our system effectively summarized user feedback in terms of user self-efficacy and third-party ratings.

## I. Introduction

Social skills are important factors that influence human life. Persistent social skill deficits hamper people from forming relationships or succeeding in social situations. Social skills training (SST), a well-established method, is a general psycho-social treatment through which people with social difficulties can obtain appropriate social skills and strengthen their social self-efficacy [1]–[2]–[3]. SST is relevant to coaching and assertion [4] and is widely used by teachers, therapists, and trainers in workplaces, hospitals, schools, etc. Bellack's method (step-by-step SST) is a structured and evidence-based SST approach that resembles a form of psycho-social training inspired by social learning theory's five core principles: modeling, shaping, reinforcement, over-learning, and generalization [5]. SST consists of the following: 1) instruction and target skills, 2) modeling, 3) role-playing, 4) verbal feedback, and 5) homework. The verbal feedback should consider a trainee's past training [5].

Automating the SST process will simplify the acquisition of such social skills by those who require them. Automated social skills training has been proposed in past researches to enhance human social skills as a substitute for human training. Some studies automated several SST steps [6]–[4]–[7], including role-playing, measuring, and feedback [8]. This automation process was done through embodied conversational agents or human-robot interaction [9]–[10]–[11]. Other works focused on improving user social skills [12] and the critical features to correctly measure them [13]–[14]–[15]. We previously developed a system that incorporated an automated social skills trainer that completely adheres to Bellack's training model through an embodied conversational agent [7]. Based on extracted multimodal features, this system provides feedback for improving the social skills of users. Experimental evaluation with graduate students showed that a larger training effect was found with our system than with control groups. These control groups received such traditional coaching as reading about social skills training and watching videos. Other work argued that people are more comfortable talking with virtual agents than strangers [12] and described the improvement of users’ social skills through subjective ratings by third parties [12]–[4]. Some research identified the effect of visualized summary and online feedback [8]–[6].

In addition, using such systematic training for people who need to improve their social skills exploits the following criteria: 1) such people favor computerized environments because they are predictable, consistent, and free from social demands; 2) they can work at their own pace and level of understanding; 3) training can be repeated until the goal is achieved; and 4) interest and motivation can be maintained through computerized rewards [16].

In summary, although human social skills training strengthens social self-efficacy and human SST by verbal feedback with considering past training history [1]–[5], no prior works have investigated the self-efficacy of users or analyzed which type of summary feedback is more effective for automated SST [6]–[8]. In this paper, we implemented a new training system called an automated social skills trainer+ and analyzed the following three hypotheses:

1) Our developed system influences users’ self-efficacy, which is related to autistic traits.
2) Third parties (human social skills trainers) can identify the improvement of learned skills.
3) Visualized multimodal feedback and training history are useful summaries of feedback types.

## II. Materials and Methods

We implemented an automated social skills trainer+ named TAPAS through the Greta Platform [17] and evaluated our system with a user study.

### A. Automated Social Skills Trainer+

**FIG. 1.**
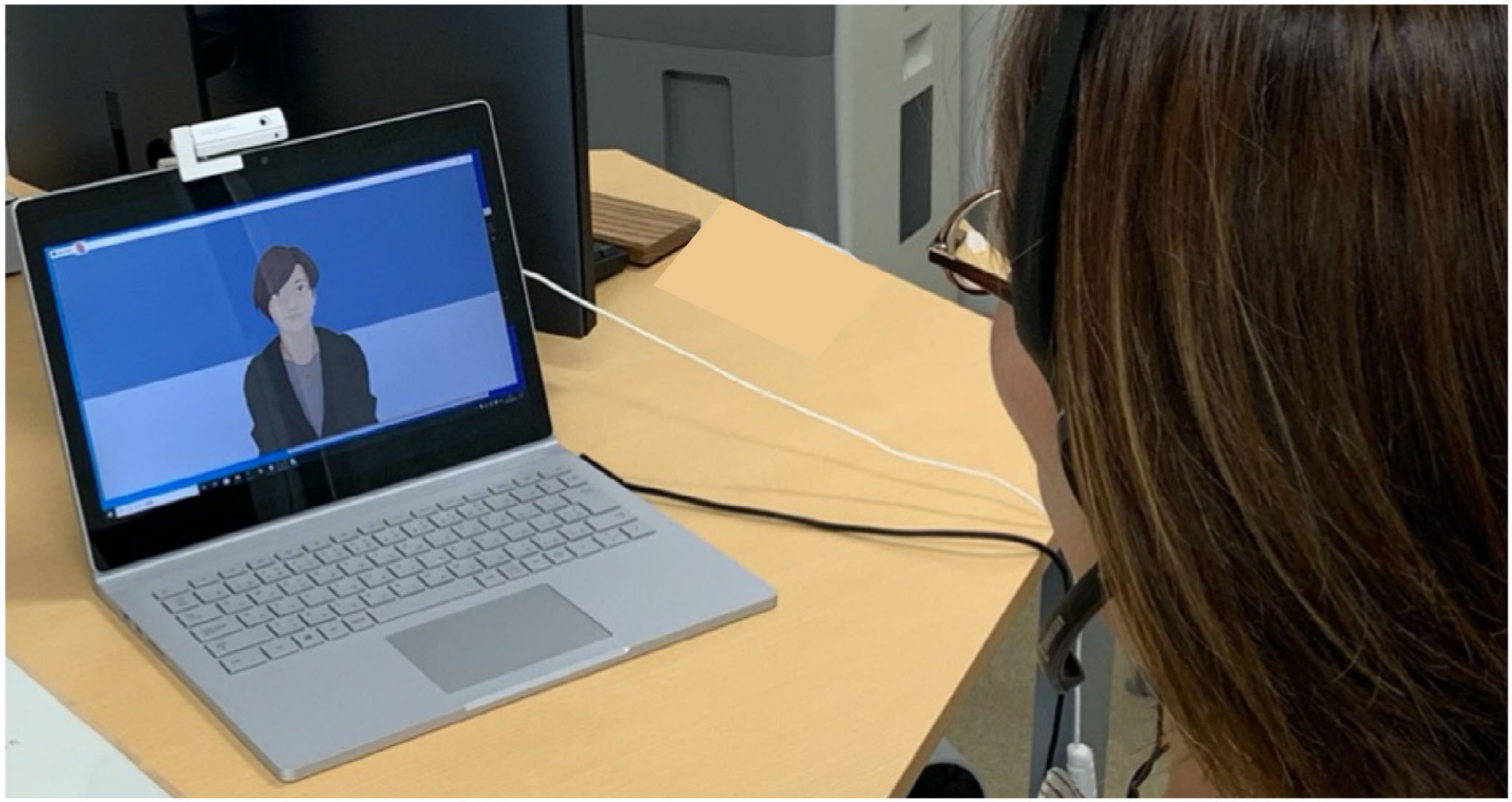
Interacting with our system.

We used embodied conversational agents (virtual agents) [17] and implemented a Japanese spoken dialogue system that integrates the following: Google speech recognition, dialogue management, CereProc TTS (voice of a Japanese woman named Yuki https://www.cereproc.com/de/node/1185), and behavior generation. We created head nodding for the behavior generation. The connection among modules was done by ActiveMQ. This system works in real-time as a Windows application. We created a female anime-type character because we learned that Japanese adults preferred such characters over more realistic ones (Fig. 1) [18].

One advantage of our system is that people can repeatedly use it at their own pace [16], unlike training with a human. The automated social skills trainer+ consists of the following elements: 1) self-introduction, 2) instruction and motivation, 3) modeling, 4) role-playing, 5) feedback and 6) homework and closing. We selected speaking skill as a target from the basic four skills [5]. Detailed scenarios were created by three psychiatrists who have SST experience (Table 1). The modeling, which was done by watching the recorded videos, includes two types: three videos of bad modeling and five videos of good modeling. Each video was selected from those used in previous works [7]–[19]. For role-plays, users recounted recent fun stories to share with the agent for about one minute. During this time, the agent nods to provide positive online feedback. Once the agent recognizes the end of a user's spoken sentence, the agent nods its head. This is created based a previous work [7]. During role-plays, the system extracts their multimodal features by a snack speech sound toolkit (http://www.speech.kth.se/snack/) for speech processing, Openface (https://github.com/TadasBaltrusaitis/OpenFace) for image processing, and Google speech recognition for speech recognition. Since the human SST did not use a complex rubric for speaking assessment (e.g., focusing on two or three important multimodal features) [5], we simply extracted five features.

**TABLE 1:** Virtual Agent Dialogue Flow

	System utterances
Self-introduction	Hello. I'm TAPAS, what's your name?
	Hello, [User name]. How are you?
Instruction and motivation	Today we are going to practice speaking skills.
	Why are they so important?
	Thank you. When you are having difficulty, you tend to overly focus on yourself.
	You might increase your strength with others by telling them what is appropriate.
Modeling	First, let's watch some videos. Pay attention to the speaker.
	Did they speak well?
	How could they speak better? I see [point].
	When talking, the important points are looking straight ahead, speaking with a smile, talking loudly, the number of words, and their difficulty.
	What you said [point] is also important.
	Let's look at some more videos. Focus on the speaker.
Role-play	Now, let's do a role-play.
	Please tell me about a recent fun story for one minute.
Feedback	Our time is up. Thank you.
	Please give me a moment to calculate your result.
Homework and closing	Try to use the skills we practiced today in your daily life.
	Thank you very much. See you next time.

Brackets denote user utterances, which were pre-processed to extract nouns.

#### 1) Summary Feedback

After the role-play, we processed the extracted features to generate feedback, which consists of these four parts:

• **User video:** Users can watch the recorded video and audio of their own talking.
• **Overall score:** We extracted the following features based on previous works: words per minute, amplitude, words over six letters (Hiragana in Japanese), and the smiling ratio in the total frames (by action unit presence: 6+12 with down sampling from 30 to 5 frames per second) [7]. We calculated a complete score based on the differences with the recordings of five good model videos [12] that compared z-scores by subtracting five times the z-score values from each feature. The maximum score is 100. That means a greater difference from the model persons indicates a smaller score. This calculation was empirically determined through discussions with a psychiatrist.
• **Comparison with models and past training:** The system uses a radar chart to compare the extracted features of the user's current speaking with that of the model person's in terms of a z-score, which is a statistical measurement of a score's relationship to the mean in a group of scores. The users were asked to completely emulate the model. We also visualized a past training by adding a different colored radar chart to the original radar charts.
• **Comments:** The system generates positive comments that reinforce the user's motivation based on features whose values are the closest to those of the models. It also generates comments about points that need improvement based on a feature that has a median distance from the models. Comments are also based on previous training. This algorithm is rule-based. If the best feature is [Feature name] in two consecutive trainings, the positive feedback notes that [Feature name] is a strength. If weak points are improved, the positive feedback notes that [Feature name] improved and provides ordinal positive comments. For example, if the feedback of the first role-play is “Try to increase your smiling ratio next time!”, then the second feedback notes that “Your smiling ratio improved and the number of words is also very good” or “The number of words is your strength.”

In this study, we implemented two types of feedback: 1) summary feedback and 2) feedback that builds on the users’ past training. For the former type, we prepared two settings: method 1: verbal comments (read by the agent); method 2: detailed parts with user videos, radar charts, scoring, and comments, as mentioned above. We prepared two cases for the feedback based on past training history: with and without.

### B. User Study

#### 1) Experiment 1: Self-efficacy and Summary Feedback

This section describes our experimental evaluation using the implemented system as well as training done by humans. We investigated the effect on self-efficacy and the feedback types.

Seventeen healthy adults participated in our study (mean age: 21, nine females and eight males), which was approved by the ethical review board of the Nara Institute of Science and Technology (number: 2018-I-1). Written informed consent was obtained from all participants. This sample size was decided based on similar previous works [7],[8],[9],[12]. Before they began to use the system, we collected social responsiveness scale-2 (SRS) scores [20] to measure their autistic traits and self-efficacy for speaking. Because there is no existing validated self-efficacy scale that targets speaking, self-efficacy for speaking was manually developed based on a previous work [2]. The participants evaluated themselves on a scale from 0 (completely unable) to 5 (moderately able) and 10 (highly able): “How well can you talk to others?" We obtained answers to this question before and after the training and compared them by a paired t-test after confirming their normality (Kolmogorov-Smirnov test: p>0.05) and equal variance (F-test: p>0.05).

**FIG. 2.**
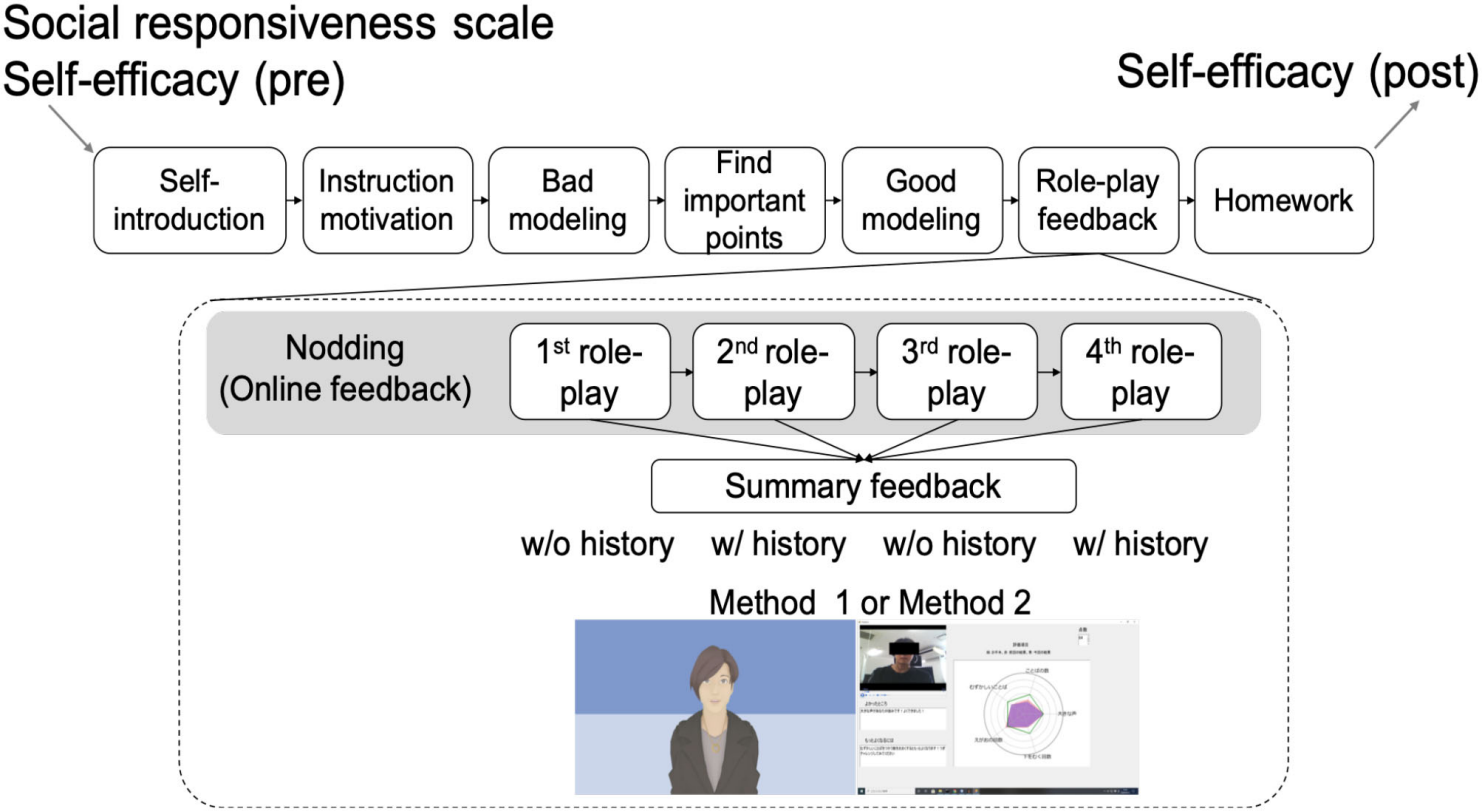
Overall system framework and corresponding experimental design.

We leveraged the automated social skills trainer+ in our experiment. The participants performed the experiment in one visit. We removed one participant who failed to complete every procedure of the experiment because he struggled to interact with the agent by voice, necessitating repeated experimenter intervention. The normal scenario used in our experiment is shown in Fig. 2 (self-introduction, instruction and motivation, bad modeling, find important points, good modeling, role-play and feedback, and homework). The participants did role-playing and received feedback four times in addition to the normal scenario. The feedback order (method 1 first: n=10 or method 2 first: n=6) was randomly determined based on simple randomization [21]. Only the experimenter knew the allocation of the feedback order. We did not tell the participants about the order or the types of feedback before the experiment (single blinding). They completed questionnaires about which type of feedback they preferred (method 1 or 2 and with or without history) by forced-choice after using the system. The questionnaires provided useful information about our overall system and its feedback (optional multiple choice). We also obtained qualitative comments regarding the feedback.

Two third-party human trainers, whose experience using and dealing with SST exceeds ten years, watched the recorded videos. They included four role-play videos from each participant. The raters were unaware of the order of the videos. We did not describe the order of the role-plays, either. The raters evaluated their overall speaking skills on a seven-point Likert scale (1: not good, 7: very good). We calculated the correlation between the scores of the two raters and averaged them. We also collected such evaluations as the story structure and the appropriateness of non-verbal behaviors, We did not analyze them in this paper because that idea is beyond the scope of this paper. We used a Wilcoxson rank-sum test to compare between first role-play and other role-plays. For all the statistical comparisons, we set the alpha to 0.05.

In addition, we examined the relationship among autistic traits, personality, and general self-efficacy by collecting their scores on the Toronto Alexithymia Scale-20 [22] and the General Self-Efficacy Scale [23]. Alexithymia is a personality trait characterized by difficulty identifying, describing, and communicating emotions [24]. To investigate the correlation between SRS and other scales, 19 healthy adults participated: ten identical (mean age: 21, six females and four males) and nine different participants (mean age: 22, four females and five males) from the original 17 healthy adults. The nine different participants did not leverage the automated social skills trainer+. The following are Pearson's correlation for SRS in nineteen healthy adults: the Toronto Alexithymia Scale, r=0.70 and the General Self-Efficacy scale, r=−0.46.

#### 2) Experiment 2: Summary Feedback With/Without Training History

Twenty-three healthy adults participated in our study (mean age: 22, nine females and fourteen males). All were different from experiment 1. The participants performed the experiment in one visit. We leveraged the automated social skills trainer+ in this experiment. The normal scenario used in it is shown in Fig. 2. The participants did role-playing and received feedback four times in addition to the normal scenario. We used verbal comments as the feedback type (method 1). The participants were randomly assigned to groups with training history (n=12) and without it (n=11). Only the experimenter knew the allocation of the feedback with/without training history. We did not tell the participants about the types of feedback before the experiment (single blinding). We used block randomization to compare two equal-sized groups [21]. Because the training included four times of role-playing and feedback, the groups with training history did receive feedback considering their history, except for the first role-play. We did not collect SRS or self-efficacy scores for speaking in this experiment.

The same third-party human trainers analyzed the recorded videos and evaluated the overall speaking skills on a seven-point Likert scale (1: not good, 7: very good). The rating was performed for only the first role-play. The raters were blinded to the groups of the participants. We averaged the scores of the two raters. The mean and SD of the third-party rating are 4.2 (SD: 1.3) for the groups with training history and 4.3 (SD: 1.4) for the groups without it (p>0.05 by the Wilcoxson rank-sum test).

After using the system, the participants answered questions designed for the lecturer criteria of the Japanese Association of Social Skills Training (http://www.jasst.net/). These criteria, which was originally created to be answered by third-party trainers, were modified to be answered by users. The question list is shown below:

1) Clarity: the feedback was clear.
2) Points I can do: I understood these aspects.
3) Points that need improvement: I understood the points that I must continue to work on.
4) Understandability: feedback was easy to understand.
5) Social adequacy
6) Agent understood me: this was asked with regard to the feedback and created by referring to a rapport agent system [25].

All of these questions were rated after using the system with a five-point Likert scale (0: not at all, 4: very appropriate). We compared each question between the groups by the Wilcoxson rank-sum test. For all the statistical comparisons, we set the alpha to 0.05.

## III. Results

**FIG. 3.**
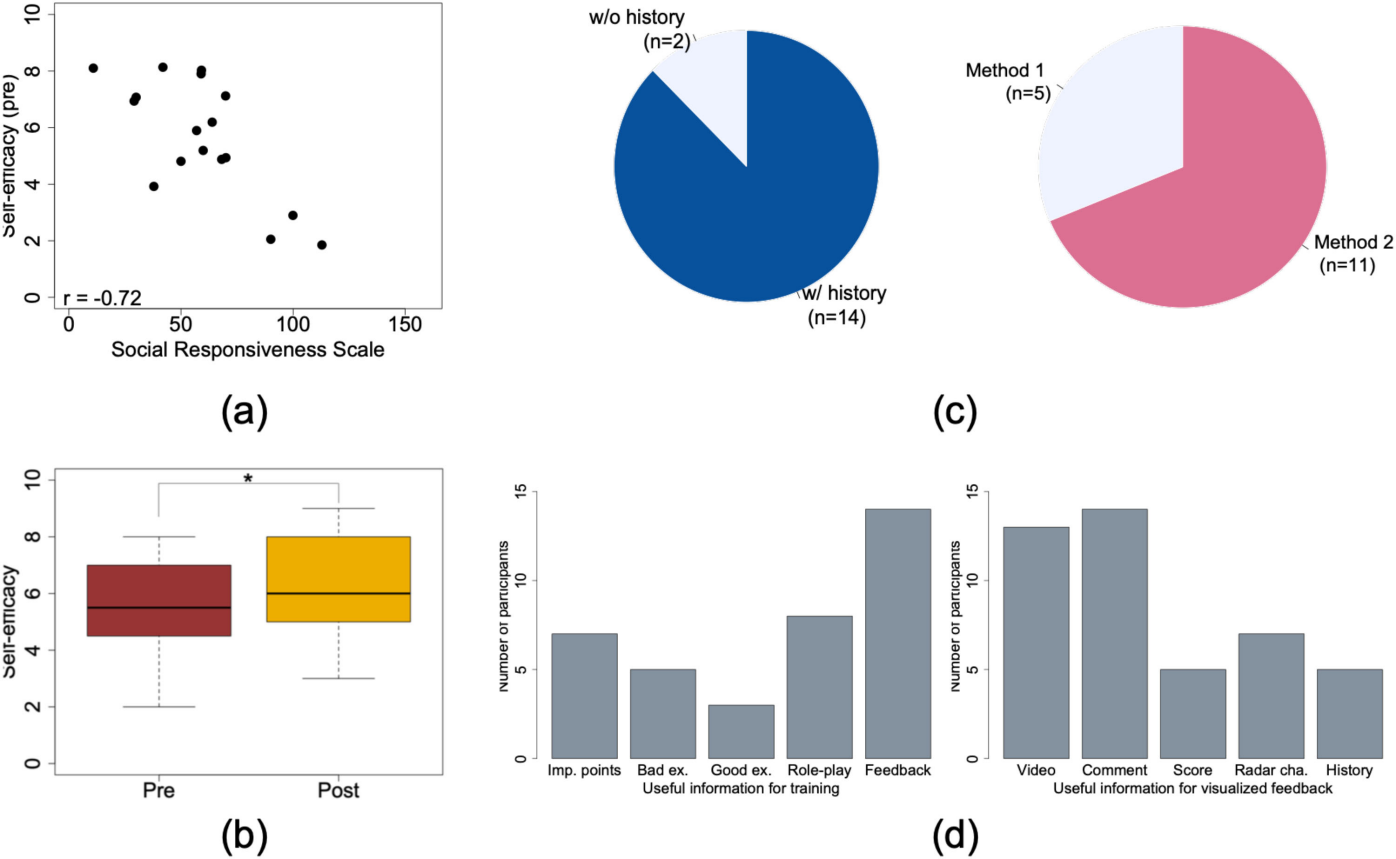
Result figures: (a) relationship between SRS and self-efficacy; (b) change in self-efficacy; (c) feedback preference; (d) useful information for training.

**FIG. 4.**
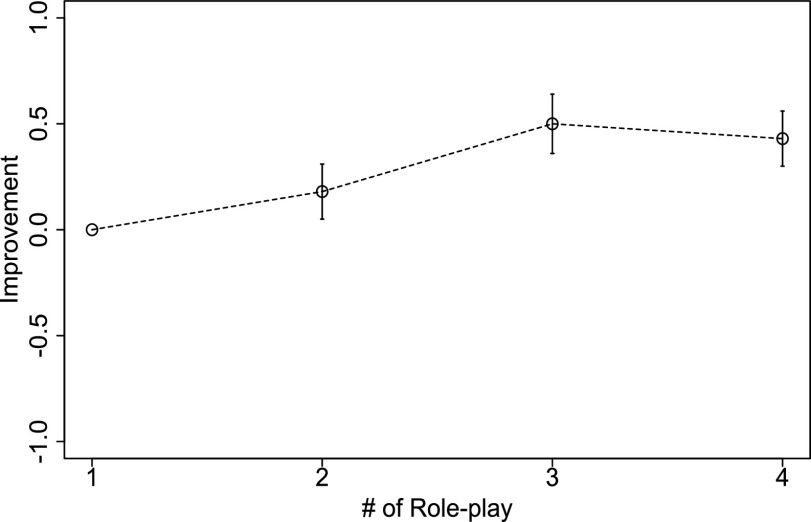
Third-party ratings with regard to overall speaking skill.

The overall results are shown in Figs. 3–4(Experiment 1) and 5 (Experiment 2).

### A. Self-Efficacy

As shown in Fig. 3(a), the results demonstrated that self-efficacy is significantly correlated to the social responsiveness scale (r=−0.72, p<0.05). Fig. 3(b) and the paired t-test show a mean improvement of 0.68 (SD: 1.08) (p < 0.05) in self-efficacy using the system. Unfortunately, the self-efficacy of two participants decreased between pre- and post-training: from 6 (pre) to 5 (post) and from 4 (pre) to 3 (post).

### B. Improvement of Speaking Skill

The correlation between the two raters was a Spearman's Rho of 0.67 (p < 0.05). As shown in Fig. 4, the overall improvement of the speaking skills in the role-plays was 0.18 for the second one, 0.5 for the third one, and 0.44 for the fourth one. Significant differences were found between the first and third and the first and fourth role-plays (p < 0.05).

### C. Types of Summary Feedback

As shown in Fig. 3(c), most participants preferred receiving feedback with history and visualized information (method 2). They gave high preference for feedback that compared their past training (14 out of 16, binomial test, p < 0.05) and method 2 (11 out of 16, binomial test, p=0.21). As shown in Fig. 3(d), they described both the feedback and the comments as very helpful. Here are two examples of qualitative comments: “By comparing the previous feedback with the new, I realized that I was consciously trying to improve.” “The radar chart was helpful because it allowed me to quickly understand my skills.”

**FIG. 5.**
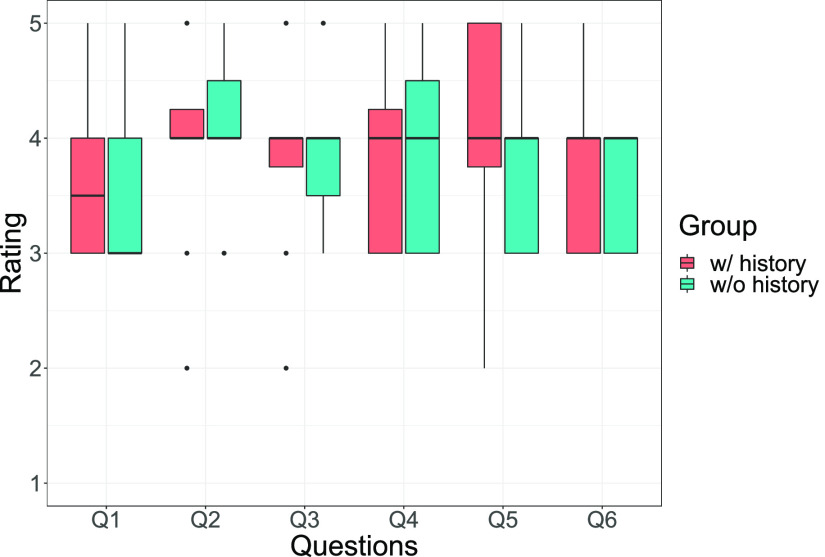
Feedback evaluation (Q1. Clarity, Q2. Points I can do, Q3. Points that need improvement, Q4. Understandability, Q5. Social adequacy, and Q6: Agent understood me).

In contrast, as shown in Fig. 5, not all the questions regarding the feedback were significantly different between the groups (all questions, p>0.05).

## IV. Discussion

Next we discuss our system's implementation of an automated social skills trainer+ and our hypothesis.

### A. System Implementation

This work is automating social skills training. We implemented and designed our system using the Greta platform and several multimodal feature extracting tools. The system, which interactively talks with users, is composed of such elements as self-introduction, observations of three bad models and five good models, role-playing, feedback, homework, etc. Its technical challenges include role-playing and feedback. Since the present study was conducted with neurotypical participants (without autism spectrum disorders), training with multiple modalities was simplified. Of course, using multisensory information for training participants with autism spectrum disorders is more complicated because they might feel overwhelmed by the quantity and the multisensory nature of the stimuli/motor skills [7]. While role-playing, the agent nodded her head after the user spoke. This action should be modulated based on imitating human SST trainers in terms of timing and appropriate empathic online feedback [26]. We created a female anime-type character, although it can be changed based on user preference.

The system leverages some elements of social skills training that have been widely studied (role-playing, feedback), but also elements that have received less attention in computerized applications (modeling, homework). We believe this system is closer to a human SST than some existing works [7]–[12] in terms of its entire dialogue scenario. For example, previous works failed to include any examples of bad modeling. With only the modeling, some participants seemed to understand the skills by comparing the bad and good examples (Fig. 3(d)). We continue to investigate how to incorporate a homework element into our system because human SST coaches use it to generalize about learned skills. Homework should be checked at the beginning of SST sessions to determine whether the previous homework was completed.

### B. Self-Efficacy

We created a self-efficacy scale for speaking based on the guidelines of a previous study [2]. Self-efficacy for speaking significantly improved between pre- and post-system use. We also found that a user's initial self-efficacy score is significantly related to SRS, which is also significantly related to the Toronto Alexithymia Scale-20 and the General Self-Efficacy Scale. Our self-efficacy scale appropriately evaluated speaking difficulty and its strengthening. Although our sample size was based on similar previous works [7], [8]–[9]–[12], further validation is required by evaluating reliability and validity with more participants.

### C. Improvement of Speaking Skill

Third-party experienced social skills trainers watched the recorded videos and rated them. The skills of the participants significantly improved after they finished the three or four role-plays and received feedback. The improvement's range (about 0.5) resembled our previous work [7]–[12]. Engaging in three role-plays is sufficient because human SST coaches generally just do one or two of them. Excessively repeating a role-play sometimes causes redundancy in neurotypical participants.

### D. Types of Summary Feedback

The training history was significantly helpful (Fig. 3(c)). However, in our algorithm, the training with history sometimes produced identical feedback as without any training, e.g., where the user did not improve a feature that he/she received and the best feature was also changed. Training history may be related to a personalized system (remembering a user's past training), and it should be validated in long-term system usage. Although summaries of audiovisual feedback are effective for most participants, we must consider a combination of both verbal comments (method 1) and audiovisual feedback (method 2). One limitation of experiment 1 was that it featured several types of feedback in one training. Experiment 2 included a single type of feedback to be trained in a randomized controlled trial. However, there were no differences between the cases with and without training history. Perhaps the difference is miniscule compared to the impact of receiving any feedback itself.

## V. Conclusion

This work's goal is automating social skills training. Our results demonstrated that self-efficacy is significantly correlated to the social responsiveness scale. Our system strengthens self-efficacy and improves speaking skills. Our participants gave high preference to feedback that compared their past training and displayed summary feedback compared to cases without such comparisons. Although summaries of audiovisual feedback are effective for most participants, we must address a system that combines both verbal and audiovisual feedbacks. This system can be integrated in the future with such other social skills as listening [19] and trained with long-term usage by considering personalized training history as humans do. We will evaluate our next system compared with human SST coaches in terms of the same evaluation criteria: third-party ratings and self-efficacy.
